# Influence of body mass index in revision total knee arthroplasty

**DOI:** 10.1590/1413-785220152306140199

**Published:** 2015

**Authors:** Rogério Teixeira de Carvalho, Diego Benone Santos, Victor Chammas, Lucas Simões Arrebola, Mauricio Lebre Colombo, Caetano Scalizi

**Affiliations:** 1 Hospital do Servidor Público Estadual (HSPE), Orthopedics and Traumatology Service, São Paulo, SP, Brazil.

**Keywords:** Osteoarthritis, knee, Arthroplasty, replacement, knee, Review, Body mass index

## Abstract

**OBJECTIVE:**

: To evaluate the influence of the body mass index (BMI) on the functional assessment of patients who underwent revision total knee arthroplasty (RTKA).

**METHODS:**

: Thirty patients who un-derwent RTKA between January 2008 and January 2012 were retrospectively assessed using the WOMAC questionnaire. The patients were divided into three groups according to the BMI ca-tegories defined by the World Health Organization (WHO): Group I with normal BMI (18-24.9 Kg/m^2)^, with eight patients; Group II, overweight (BMI 25-29.9 Kg/m^2)^, with 15 patients, and Group III obesity with BMI ≥ 30 Kg/m^2^, with seven patients. The post-ope-rative function scores obtained through the WOMAC questionnaire were compared with the BMI of each group. The statistical analysis between BMI and WOMAC scores was performed with the Spe-arman correlation test.

**RESULTS:**

: The average functional WOMAC score for individuals in Group I was 16.7; in Group II it was 47.7; and in Group III it was 69.9, with a statistically significant differen-ce between groups I, II and III (p< 0.0001).

**CONCLUSION:**

: Patients with BMI > 25 Kg/m^2^ had a worse functional evaluation through WOMAC scores when compared to patients with normal BMI after RTKA. **Level of Evidence III, Tranversal Retrospective Study.**

## INTRODUCTION

Osteoarthritis (OA) is a progressive musculoskeletal disorder, which typically affects the joints of the hands, spine, hip and knee. It is the most common joint disorder and may affect between 6% and 12% of the adult population and more than 30% of people over 65 years of age.^1^

The knee joint is one of the main areas affected by OA and it is present in about 6% of the adult population over 30 years old, and its prevalence increases to 10% in people over 55 years of age.^2^

It is considered a disease with multifactorial etiology and some aspects such as older age, female gender, obesity, anatomical deformities, joint damage and certain professional activities are important risk factors for the emergence or worsening of knee OA.^3^ Regarding these factors, obesity is one of the most significant and foreseeable risk factors for the development of osteoarthritis, because of the increased mechanical load on the cartilage and subchondral bone.^4^

According to the World Health Organization, a body mass in-dex (BMI) range considered normal is 18-24.9 kg/m^2^, overwei-ght 25.0 - 29.9 kg/m^2^ and obesity BMI equal to or greater than 30.0 kg/m^2^. The index is calculated by the ratio of the individual's weight (in kg) by the square height (in m). Yeung et al.^5^ showed that obese people (BMI ≥ 30) suffer a higher incidence of complications, have lower rates of implant sur-vival and lower scores function after total knee arthroplasty (TKA). The result of the WOMAC score after TKA in patients with high BMI is lower than in population with normal BMI.^6^ A high BMI (≥ 25 kg/m^2)^ has a negative effect on functional outcome after total knee arthroplasty procedure.^7^ The incre-ased proportion of obese population (BMI ≥ 30 kg/m^2)^ com-bined with an increased demand for TKA will inevitably lead to an increase in the number of obese patients candidates for TKA and consequently the total knee arthroplasty revision surgeries (RTKA).^8^

The RTKA has as main etiologies septic and aseptic cau-ses.^9^ Among the most common causes of aseptic TKA re-vision surgeries, we highlight the release of the prosthetic components, polyethylene wear, osteolysis and periprosthetic fracture. Some studies show worse postoperative functional WOMAC outcomes score in revision procedures compared to primary arthroplasty, as well as worse postoperative scores in septic revisions compared to aseptic revisions.^9^ There are few studies in the literature that evaluate the function after RTKA in relation to BMI. The hypothesis of our study is to assess whether patients with BMI ≥ 25 kg/m^2^ undergoing RTKA have worse functional WOMAC scores when compared to subjects with normal BMI subjected to the same procedure.

The objective of the study is to evaluate the influence of BMI on the functional evaluation by the WOMAC score of patients undergoing total knee arthroplasty revision surgery.

## METHODS

We retrospectively evaluated 30 patients undergoing RTKA at the Orthopedic Service between January 2007 and January 2013. The study was approved by the Ethics Committee of the Institution under number 404 694. All patients signed a Free and Informed Consent form.

The mean age of the patients was 75.7 years old (range 60-87 years old). Regarding gender distribution, eight patients were male and 22 were female. The average time between the first TKA surgery and RTKA was 72 months (range 8-156 months). All patients underwent RTKA by the same surgeon, a specialist in knee at the Orthopedics Service, with the same revision prosthesis implant (TC3(r) DePuy, Warsaw, IN, EUA). The postoperative physical therapy protocol up to 6 months was the same for all patients, and was performed by the same team of professionals.

Inclusion criteria were: literate patients (minimum three ye-ars), submitted to two or three surgical procedures (including TKA and RTKA), walkers for the activities of daily living with or without orthoses. Exclusion criteria were: patients who underwent more than three surgeries, patients with metaphy-seal bone defects in the proximal tibia and/or distal femur, people with endocrine or eating disorders (anorexia, bulimia), patients with other orthopedic or neurological disorders or with active infectious processes (sepsis, pneumonia, urinary tract infection).

Each patient had his/her BMI calculated from self-reported height and weight, as judged by Dekkers et al.^10^ According to the World Health Organization (WHO), normal BMI ranges 18-24.9 kg/m^2^, overweight 25.0-29.9 kg/m^2^ and obesity, equal to or greater than 30.0 kg/m^2^.^11^ In the sample, patients were divided into three groups: Group I had normal BMI (eight patients), Group II were overweight (15 patients), and Group III, obese (seven patients).

Data were collected retrospectively, and the score of postope-rative WOMAC function (Western Ontario and McMaster Uni-versities Osteoarthritis Index) assessed by a single experien-ced examiner, after 6 months of RTKA, was used to compare the groups. The WOMAC scoring system, originally published in English, was used to determine the individual's function after knee and hip replacement procedures, and has been translated, validated and adapted for Portuguese language.^12^ The WOMAC score assesses 23 items divided into three subs-cales (pain, stiffness and function) which are combined to produce an overall measure of the individual's health. Each item receives a score ranging from zero to four, the highest scores denoting a worse functional score.

The WOMAC function score does not define values ​or value ranges to stratify patients interviewed on satisfactory or unsatisfactory function. Thus, the score provides a comparison of groups subjected to RTKA.^12^

Statistical analysis of BMI data and the etiology of RTKA with the WOMAC score was performed by the Spearman correla-tion test and statistical significant was set at p < 0.05.

## RESULTS

**Figure 1:**
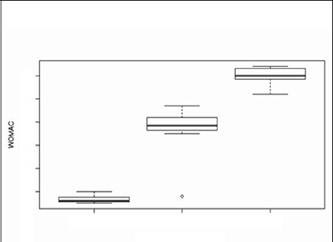
. Relationship between BMI and WOMAC

The mean WOMAC score in Group I was 16.7 (range 15-20). In groups II and III the mean WOMAC score was 47.7 (range 18-57) and 69.9 (range 62-74), respectively. When comparing WOMAC score of the three groups analyzed, we observed that the patients with BMI equal to or greater than 25 kg/m2 (Groups II and III) had statistically significant worse functio-nal scores than the group of eutrophic patients (Group I), (p <0.0001). Figure 1 shows a box plot that demonstrates the variability between these groups. In Group I, the upper and lower limits corresponded respectively to 15 and 20, with 50% of patients in this group up to 16.7 and a very small variation (standard deviation 1.7). In Group II, lower and upper limits of 45 and 57 were observed, respectively, with 50% of patients up to 47.7 and a standard deviation of 8.6. In this group we observed the presence of an outlier point, with a value of 18. In Group III, the upper and lower limits are, respectively, 68 and 74, with 50% of the patients with 69.9 and a standard deviation of 4.1. (Figure 1)

**Figure 2:**
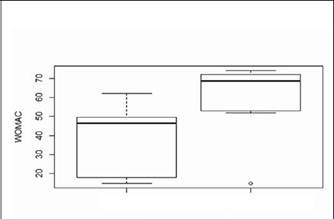
. Relationship between RTKA etiology and WOMAC. AG: Aseptic etiology group of total knee arthroplasty revision surgery (RTKA), SG: Sep-tic etiology group of total knee arthroplasty revision surgery (RTKA)

The patients were also divided into two subgroups regarding the etiology of RTKA. Of the total sample (30 patients), 10 patients had septic etiology of these RTKA and in these pa-tients the average WOMAC functional score was 60.4 (range 15-74) and 20 patients had aseptic etiology of RTKA, with functional WOMAC average score of 38.3 (range 15-62). Thus, we observed that patients undergoing septic etiology of RTKA had worse functional WOMAC scores than the group who underwent aseptic etiology of RTKA (p-value = 0.001). Figure 2 shows the relationship between the variables, etiology and WOMAC. In the aseptic etiology group (AG) we observed a large variation in WOMAC values among individuals (SD = 16.4) around the mean of 38.3 (AG bold line) with minimum 15 (AG baseline) and maximum of 20 (AG topline). In the septic etiology group (SG) there was also a wide variation of WOMAC values between​ individuals (DP = 18) around a mean of 60.4 (SG bold line). (Figure 2)

In Group I, only one patient underwent RTKA due to septic cause, with functional WOMAC score of 15, while the remai-ning seven patients underwent reoperation due to aseptic loosening of prosthetic implants and mean WOMAC of 17.1 (range 15-20).

In Group II, three patients underwent RTKA due to septic cau-se, practical WOMAC score of 54, while the remaining 12 patients underwent reoperation due to aseptic loosening of prosthetic implants, mean WOMAC of 48.6 (range 45-55).

In Group III, only one patient underwent RTKA for aseptic cau-se, with WOMAC functional score of 62, while the remaining seven patients underwent reoperation due to septic loosening of the prosthetic implant, with an average WOMAC of 71.1 (range 68-74).

Regarding complications, in Group III one patient had a car-diac event that required transfer to the intensive care unit (ICU) in the immediate postoperative period. In Group II, one patient developed deep vein thrombosis (DVT). Another was transfer-red to the ICU for pneumonia. In Group I there were no com-plications. All patients performed well regarding ambulation, with no significant functional deficits, reoperation or death.

## DISCUSSION

The influence of BMI on the functional evaluation after RTKA surgery is little studied in the world literature. Previous sys-tematic reviews reported, in the medium term, a higher rate of failure, complications and lower scores function in obese patients undergoing RTKA.^13^ However, the effect of BMI on functional outcomes was not specified.^14^ Our study showed that the patients achieved different levels of function after RTKA depending on postoperative BMI values. Patients with BMI equal to or above 25 kg/m^2^ had lower function levels ba-sed on WOMAC score as compared to patients with normal BMI subjected to RTKA procedure.

Regarding the etiology of RTKA, in several studies the results of septic revisions have been worse when compared to aseptic revisions.^15^ Ghanem et al.^15^ observed a worse WOMAC score in the septic group as compared to the aseptic group after two years of follow-up. Although it was not the focus of the study, we found similar data from the literature, with worse WOMAC function scores in patients undergoing septic revision.

Infection after TKA occurs more frequently in obese patients.^16^ In our study, six patients among 10 who underwent septic etiology of RTKA were obese. These patients have more co-morbidities and higher rates of complications than non-obese patients, which negatively influence the functional outcome after RTKA in these individuals.^16^

Studies showed that many factors are associated with func-tional outcome after TKA, but not all have been identified, none is individually decisive, enduring the controversy over its impact.^17^ For patients with knee OA who are obese, seve-ral anthropometric characteristics of lower limbs, the degree of intraoperative difficulty and postoperative complications negatively influence the WOMAC postoperative score.^18^ The identification of these factors is assumed to be associated with worse functional outcomes after TKA and RTKA in this group could help select which patients need additional measures preoperatively or during surgical approach.

Although it is postulated that BMI influences on postoperative function of the patient, we advise caution in interpreting the data presented here. Several factors including mental health status and depression, general health status, the need for multiple surgeries and patient expectations are all known to influence the patient's satisfaction.^19^ ^20^ Since it was not pos-sible to measure and adjust all these factors, these variable can be a source of confused interpretation.^19^ ^20^ We found in our study a relationship between BMI equal to or above 25 kg/m^2^ and worst functional WOMAC outcomes as compared to subjects with normal BMI underwenting RTKA.

Regarding complications after RTKA which negatively influen-ce the functional outcome, there was a patient in the obese group that had a cardiac event that required transfer to the intensive care unit (ICU) in the immediate postoperative pe-riod. In the group of patients classified as overweighed, one patient developed deep vein thrombosis (DVT) and another was transferred to the ICU for pneumonia. In the group of patients with normal BMI, there were no complications influen-cing the functional outcome.

The main limitations of this study were: BMI was calculated from patient's self-reported height and weight, which does not have the same accuracy of objective measurements. However, a previous study showed that self-report may be used in as-sessing the patient's height and weight in both overweight and malnutrition in the obese population. 10 Moreover, the follow-up time was only short term, and the causes of revision surgery within aseptic etiology RTKA group were not discriminated. Future studies would evaluate the influence of etiology on the functional assessment after RTKA.

Our study did not assess patients who underwent more than three TKA. However, the influence of comorbidities in the early postoperative period, readmissions due to complications, hospital costs involved, the severity of complications, quality of postoperative life, death rate and durability of the implants have not been evaluated in this study and may impact the function af-ter RTKA, making new studies in this area of prime importance.

## CONCLUSION

This study has demonstrated that patients with BMI equal to or above 25 kg/m^2^ had poorer functional assessment by the WOMAC score as compared to patients with normal BMI after revision total knee arthroplasty. 
